# Comparative analysis of country-level enablers, barriers and recommendations to strengthen institutional capacity for evidence uptake in decision-making

**DOI:** 10.1186/s12961-020-00546-4

**Published:** 2020-07-09

**Authors:** Meike J. Schleiff, Alice Kuan, Abdul Ghaffar

**Affiliations:** 1 Bloomberg School of Public Health, Johns Hopkins University, 615 North Wolfe Street Room E8638, Baltimore, United States of America; 2 Johns Hopkins University, 3400 North Charles Street, Baltimore, MD United States of America; 3 Alliance for Health Policy and Systems Research, 20 avenue Appia, Geneva, Switzerland

**Keywords:** Evidence uptake, health policy and systems research, research to practice, capacity strengthening

## Abstract

**Background:**

Evidence-based decision-making is crucial to leadership in the health sector to identify country-level priorities and generate solutions supported by rigorous research. Barriers and enablers have been explored, but limited evidence about what works to strengthening capacity at individual and institutional levels within countries has been reported, and inconsistent use of evidence to inform policy-making is a persistent challenge and concern.

**Methods:**

We conducted a framework analysis comparing experiences of nine purposively selected countries (Chile, Ethiopia, Ghana, Kyrgyzstan, Lebanon, Mozambique, Rwanda, South Africa and Sri Lanka). We utilised qualitative case studies developed by in-country teams to explore enablers and barriers described across components of a predefined theory of change and then identified six cross-cutting themes and recommendations for relevant stakeholders associated with each theme.

**Results:**

The cross-cutting themes included (1) leadership and political will, (2) incentives and resources, (3) infrastructure and access to health data, (4) designated structures and processes, (5) interaction and relationships, and (6) capacity strengthening and engagement. While each case country’s context and experience was different, common enablers and barriers surfaced across each of these themes, with Ministries of Health and other government agencies having strong roles to play, but also recognising the need for other stakeholders, including researchers, donors and civil society, to serve as essential collaborators in order to strengthen evidence uptake. Substantial and sustained investment in research capacities, able leaders and stronger engagement of civil servants are needed to further this progress and strengthen processes of health decision-making.

**Conclusions:**

All countries represented in this study have made commendable progress in increasing evidence uptake and strengthening supportive systems. Establishing and strengthening necessary structures and the relationships that underpin them takes time as well as resources. Going forward, the findings from this study can help guide and support advocacy to increase domestic funding for health research, especially health policy and systems research, and ensure that civil servants as well as researchers have the capacity and support to collaborate and continue to bolster evidence uptake.

## Background

The need for research uptake in decision-making processes of Ministries of Health (MoHs) and other health authorities as well as among civil servants (CSs) has been acknowledged for some time. Several reviews and a number of case examples have pointed towards the facilitators and barriers for effective evidence-based decision-making, especially within a particular organisation or project. Two systematic reviews have also looked at aspects of use of evidence by policy-makers, including barriers such as mistrust between researchers and policy-makers, power and budget struggles, poor access to research, and a lack of timeliness and synthesis of evidence, and enablers such as personal contact, building relationships, timeliness of evidence, and summaries of evidence with policy recommendations [1, 2]. However, to date, there is limited literature available that analyses national case examples and looks across contexts at existing levels of capacity or provides experienced and evidence-based recommendations about how best to support countries to increase research uptake. Case studies, such as experience from Canada, exist where sustained effort and investment has gone into establishing centres, training programmes and processes for evidence translation and use [3, 4]. However, globally across high-income as well as low- and middle-income countries, inconsistent use of evidence to inform policy decisions persists as a challenge and limitation and there is limited guidance available in the literature about how to address this.

A broadly accepted definition of capacity in the context of development work is “*the ability of individuals, institutions and societies to perform functions, solve problems, and set and achieve objectives in a sustainable manner*” [5]. ‘Capacity’ at the institutional level can be developed through approaches that affect the system more amply, such as processes that are sustained over time and not as easily derailed by changes in individual staff, or structures that ‘institutionalise’ these processes across a wide range of decision-making stakeholders [6]. ‘Capacity’ therefore goes beyond a mere technical issue; at the individual level it is also one of attitudes, motivation and ability to assume a desired behavior while at the institutional level it also relates to governance structures and mechanisms as well as incentives [7].

The field of health policy and systems research (HPSR) has seen rapid expansion in recent years and continues to evolve. HPSR is often characterised by the research questions that are asked, regardless of the discipline or focus that researchers might come from [8, 9]. HPSR may draw upon disciplines such as epidemiology, policy analysis and economics, among others; however, the research questions dictate the study design and methodological and analytical approaches used. In addition, HPSR is often intended to respond to immediate practice concerns and generate lessons that are more immediately applied to health policy and systems issues. HPSR often includes high levels of collaboration with diverse stakeholders and the need to engage directly in the translation of evidence into practice, leadership and advocacy [10]. A recent mapping study of global HPSR capacity strengthening demonstrated a range of training opportunities as well as significant gaps in terms of geography, scope and modalities [11, 12]. Recent literature has emphasised the need for strengthened capacity for “*data systems and institutional capacity to use and manage data for effective decision-making*” as a foundation for achievement of global goals such as the Sustainable Development Goals and Universal Health Coverage [13]. Further, evidence-based decision-making does not take place in a depoliticised vacuum. Political alliances and priorities, knowledge brokers [14], and other contextual factors have a substantial role to play.

The purpose of this study is to analyse nine purposively selected case studies of national MoHs and other key stakeholders that they work with to identify and describe enablers and barriers to evidence uptake across countries, within the complexity of the contexts in which they are experienced. We aim to provide recommendations on how key stakeholders, including policy-makers, researchers and development partners, can contribute to strengthening capacity for the generation and use of evidence, and in particular HPSR, to inform decision-making at the country level.

## Methods

We conducted a framework analysis in order to obtain a holistic understanding of the evidence uptake case studies that we analysed and to identify cross-cutting themes across cases. A framework analysis is appropriate when the desire is to systematically analyse a set of similar kinds of data (i.e. with the same themes or topics so that it can be consistently categorised) [15]. Framework analysis can facilitate the identification of key themes using a matrix approach to compare data and extensive memoing [16] and discussion among research team members to identify linkages between themes and key messages of interest [15].

### Case study selection and development

**Fig. 1:**
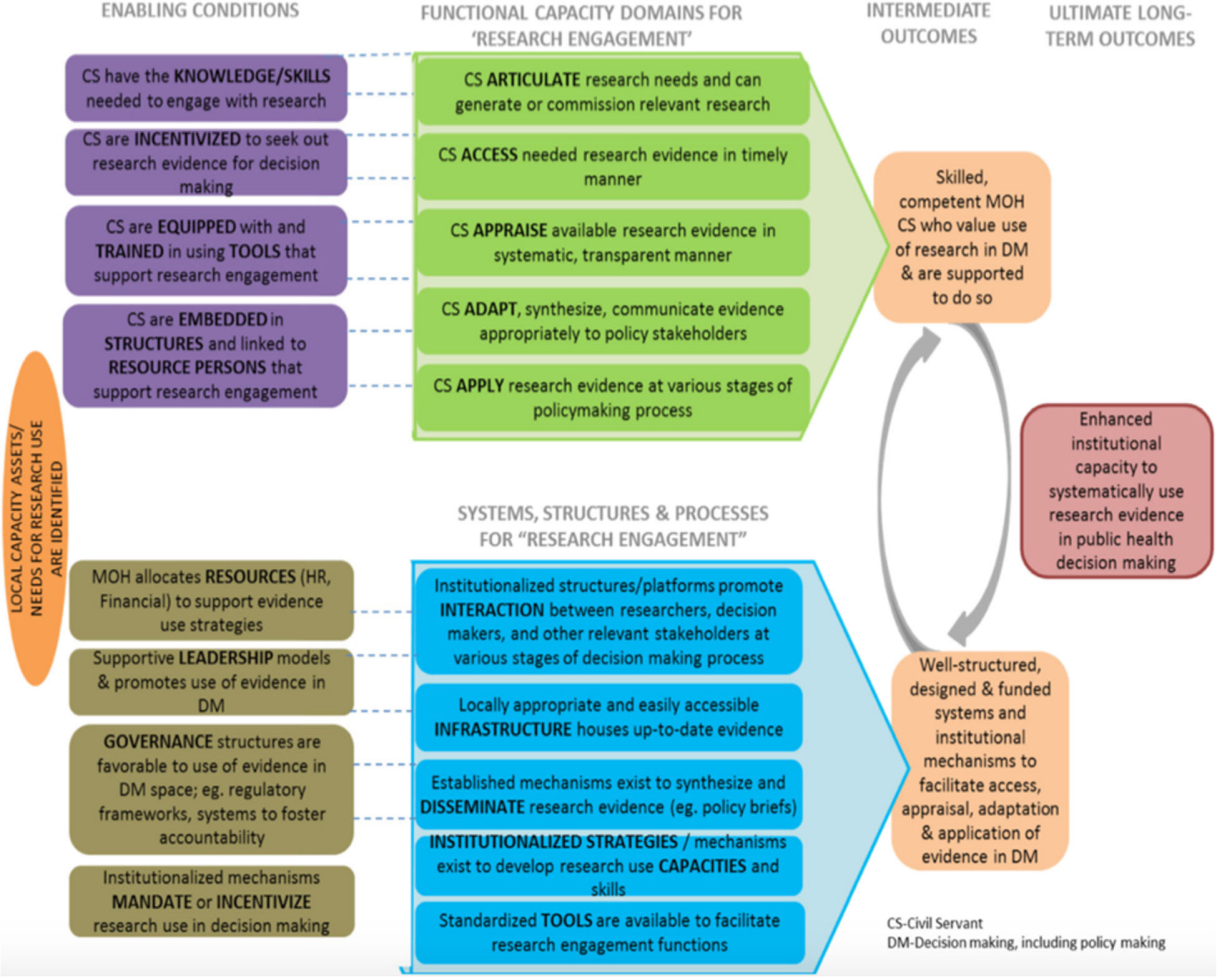
Theory of Change for enhancing capacity of decision-makers for evidence uptake [17, 18]. This figure was utilized by the Alliance for Health Policy and Systems Research when they issued their HIGH-RES call for proposals to develop the original case studies analysed for this paper [17]

Countries to serve as case examples of how institutional capacity has been built were selected purposively by the Alliance for Health Policy and Systems Research leadership to capture experiences across high-, middle- and low-income contexts. The first section of the Additional file associated with this article provides further details about the contexts of each of the case countries. A key consideration in case country selection was to be able to explore different ways of strengthening capacity for HPSR and other evidence use in decision-making. All countries that were selected — Chile, Ethiopia, Ghana, Kyrgyzstan, Lebanon, Mozambique, Rwanda, South Africa and Sri Lanka — are known for their efforts to use research to inform decision-making and have a history of work in this area. Teams within each of these countries were identified and contracted by the Alliance to prepare in-depth written case studies based on a combination of literature review and interviews with key informants to answer a set of questions related to a common Theory of Change (ToC) (Fig. 1) [17, 18]. All case study development teams were provided with a common conceptual framework, guidelines for the report development and ongoing guidance from the Alliance staff. Case studies were prepared between late 2017 and the end of 2018. For this study, we chose to utilise these existing case studies due to the purposive sampling approach that was used to select them, extensive effort of in-country teams to develop them drawing upon historical experiences, literature, key informants and other data sources, and their consistent alignment with the common ToC.

### Data cleaning and coding

In preparation for a cross-case analysis, we read all the cases in their entirety, including the methods sections and a review of bibliographic information to assess the quality and reliability of each case study’s content. Then, we removed all figures, tables, boxes and other formatting from the cases in order to conduct coding of the main textual content in Dedoose version 8.2.14. We retained copies of all content that was removed and noted in the text where it was referenced in order to ensure that we did not miss any relevant content related to each case study. We developed a codebook based on the ToC with the addition of the PESTLE (Political, Economic, Social, Technological, Legal, Environmental) analysis [19] dimensions in order to systematically explore dimensions of context that influenced the evidence generation and use process within each country context. We conducted a pilot test of coding material using two of the case studies that had slightly different structures (Ethiopia and Mozambique) in order to clarify areas of confusion and ensure that both coders (MS and AK) had a shared understanding of how to consistently code material. Several codes were added in addition to those corresponding with components of the ToC in order to enable the capture of strong illustrative examples as well as rich descriptions identified within the data.

### Data analysis

Through a series of summarisation and synthesis steps, we reviewed data for each code across country cases and developed matrices with summaries of each country’s data as well as overall summary notes for each code. During the first iterative step, we teased out enablers and barriers within each code for each case study, and during later iterations we explored linkages between codes and developing key messages and recommendations, supported by illustrative examples from the data. A set of six cross-cutting themes with corresponding recommendations were developed for presentation in this paper.

## Results

This section provides a brief comparison of key contextual features of all of the case countries. We then present our findings related to capacity strengthening for evidence use through the lens of a set of cross-cutting themes and associated recommendations that emerged from the data.

### Comparing country contexts: political stability, economic growth and social factors

Country cases analysed for this study differ across political, economic and social contexts. These contextual factors have direct effects on the trajectories of each country towards strengthening the individual and institutional capacity for the use of evidence in policy- and decision-making. The Additional file material associated with this article provides further details about the context of all nine of the case study countries. In terms of political factors, some countries had worked to decentralise their health system in recent years, while others have maintained more centralised systems and focused rather on strengthening the public sector services and building collaborations with other partners and the private sector, and/or invested in improving primary healthcare services using community health workers and new financing models. All countries faced political challenges — whether related to conflict, corruption or others — but had also been successful in maintaining some political will and focus on improving the health system and using evidence to support this goal.

Economic factors included heavy dependence on foreign aid for several countries as well as challenges in mobilising and sustaining domestic resources for health, both of which could substantially affect activities within countries’ MoHs. Countries also had diverse perspectives on payment for public sector services (primary and preventive/promotive care through to more specialised curative care) and what kinds of care received the most budget and focus within the health sector. Relatedly, social factors that were captured in the study included a range of population sizes across countries (from about 6 million in the smallest countries up to over 100 million in Ethiopia) and variance in the numbers of different ethnic groups within a country. Many countries reported large proportions of their populations still living in rural areas, though urbanisation is a simultaneous trend. Inequities in access to care as well as outcomes across ethnic and rural/urban populations were a concern in several countries.

Finally, each country had a unique trajectory in terms of how the use of evidence became a priority and how that use has evolved and strengthened over time. Post-war, or post-social reformation or revolution, several countries introduced evidence as part of a re-invention of their societies. Other countries have been incentivised and driven towards use of evidence due to donor expectations. Still others have recognised fundamental changes in population demographics and/or pressure from citizens to better understand and respond to emerging challenges. Though original motivators as well as specific approaches have varied across countries, all countries have made exceptional gains in evidence uptake over recent decades and recognise additional opportunities to continue to strengthen and expand the capacity that has been created so far.

### Cross-cutting strategies for the development of capacity for evidence utilisation

The six cross-cutting themes presented in Table 1 were derived based on the full framework analysis process. These themes are interconnected, and while we have attempted to minimise overlap and clarify the boundaries of each theme by defining each and honing these through multiple discussions among co-authors, there remain some areas of convergence and likely minimal duplication of information. For each theme, we have provided a brief description, a list of enablers and barriers identified from across the cases we analysed, and reference to the specific illustrative examples that are further described in the Additional file. Summaries of the selected illustrative examples for each of the themes are provided in this main text to illustrate how each theme is reflected in real events.

**Table 1:** Cross-cutting themes for evidence uptake and related enablers and barriers identified from case studies

Cross-cutting theme	Theme description	Enablers and barriers
1. Leadership and political will	Creation of a culture of evidence use is determined to a great degree by the skills and priorities of managers and leaders within the MoH; in addition, centres or units that are specifically created to support/utilise HPSR need adequate leadership in order to be successful	*Enablers:* ● When leaders prioritise evidence generation and use, they can motivate other staff around them to do the same ● When leaders prioritise evidence, they can institutionalise processes for its generation and use by using political commitments to set up structures to support it ● When leaders prioritise evidence use, this can also attract additional resources to support those functions
		*Barriers:* ● When leaders do not have strong agenda-setting and governance skills and appreciation for the research process themselves, they may not understand the value of evidence nor the time and resources required for staff to engage in these activities ● When leaders are focused on care delivery rather than evidence use, then that is where time and resources will be allocated instead ● When leaders are enmeshed in political processes or dynamics that are not focused on evidence (due to pace, corruption, conflict, etc.), then this can detract from overall evidence use
		*Illustrative examples (see Additional file for full descriptions):* • Ethiopia’s One Plan, One Budget, One Report • Rwanda’s results-based financing and performance-based financing strategies
2. Incentives and resources	Evidence uptake requires funding and adequate support of staff; financial resources include support for training programmes, providing higher salaries for researchers, allocating more of the MoH’s budget towards research, dissemination and use; incentives can also include a desire to impress leadership, performance requirements and other accountability mechanisms	*Enablers:* ● Opportunities for training, networking with higher-level staff, traveling for work opportunities, and new responsibilities can be motivational to CSs; this is strongly linked with whether leadership prioritises HPSR and evidence use and therefore whether they direct resources towards it ● Salary top-ups meant to promote research activities can increase evidence generation among CSs (sometimes the first priority for these programmes is to counteract the low based salaries for CSs and the evidence generation is practically a secondary priority) ● Government policies requiring a certain percentage/amount of budget allocated towards research and international declarations and goals (e.g. the Algiers Declaration, World Health Assembly resolutions on health research, and the Bamako Call to Action on Research for Health) [20, 21] ● Strong connection with DPs, who want to see stronger evidence use in decision-making, can leverage international funding sources ● Sufficient resources to invest in building/strengthening entities that support health systems research such as rapid reviews mechanisms
		*Barriers:* ● Inadequate resources (domestic resources, particularly in low- and middle-income as well as international) and/or resources earmarked for priorities that are not aligned to national strategy/needs; can result from reliance on DPs who do not prioritise (and thus do not fund) research activities ● Lack of career pathways and other incentives compared to clinical researchers and policy-makers ● Because of the time lag between the generation of evidence, uptake and related action, leaders are not always inclined to use their experience and authority
		*Illustrative examples (see Additional file for full descriptions):* • Rwanda’s increase in domestic resources and ‘district challenge fund’ • The Ethiopian Public Health Institute’s staffing evolution and opportunities
3. Infrastructure and access to health data	Infrastructure is a barrier to evidence uptake when appropriate processes and permissions are not present to enable access to needed information; this includes across agencies, ministries or partners	*Enablers:* • Ability to share data between CSs and researchers and also between different agencies and sectors with government • Data access, including real-time data, synthesised data, data from different agencies, having relevant documents and data in one place that is accessible to everyone • Digitisation of datasets, reports and processes can greatly expedite the speed of access, analysis, dissemination (as long as supporting infrastructure such as electricity, internet, functional computers are present) • Presence of a national statistics unit (not necessarily health specific) in multiple cases has also aided in data analysis and availability of digestible/usable results
		*Barriers:* ● CSs not trained on how to use available databases ● Databases not functional due to other infrastructure limitations (electricity, internet, functional office spaces) ● Language barriers to being able to review and glean relevant information from scientific/international evidence ● Incoordination and miscommunication between data units and policy-makers
		*Illustrative examples (see Additional file for full descriptions):* • Chile’s integrated health technology assessment process
4. Designated structures and processes	Focused units, centres and other platforms that are institutionalised — not relying on individual relationships — can ensure accountability and sustainability of evidence-use demand; these structures can facilitate a coherent national research agenda or strategy	*Enablers:* • National research agendas or policies established, often as part of a health systems reform processes • Focused units/centre established that have resources (finances, staff, infrastructure) to support their mandates • Local universities can be key players in these structures, when brought in strategically as partners and supported to understand and keep up with the pace of MoH evidence needs/decision-making processes • Presence of tools and guidelines (international and national) for monitoring and evaluation planning can support these structures and processes
		*Barriers:* ● Lack of focus on research/evidence use within MoH structure (make it challenging to strengthen a culture of evidence use) ● Legal or financial issues related to the autonomy/legitimacy of these structures ● Lack of designated positions and roles for CSs to engage in evidence generation/use
		*Illustrative examples (see Additional file for full descriptions):* • Mozambique’s Human Resources for Health National Directorate • Lebanon’s Knowledge to Policy Center
5. Interaction and relationships	In order for effective evidence use to take place, multiple stakeholders are involved (researchers, CSs, donors, civil society and professional associations); these stakeholders need opportunities to interact with each other in order to share knowledge, understand priorities and build trusting and effective relationships	*Enablers:* • Many stakeholders can contribute to this interaction in a constructive way, including beyond just CSs and researchers • Pre-established policies or regular events (face-to-face, if possible) that bring researchers, CSs and policy-makers together with accountability of all parties; this includes national meetings or mandates that policy decisions must be backed by sufficient evidence • International organisations or declarations/targets can help provide stability and another perspective during dialogues in order to break through political stalemates or ‘old’ patterns
		*Barriers:* • Building trusting and functional relationships among stakeholders takes time • Lack of supportive infrastructure, including communication pathways, meeting spaces and leadership of these interactions can result in frustration and/or lack of results
		*Illustrative examples (see Additional file for full descriptions):* • Kyrgyzstan’s approach to responding to rising levels of cardiovascular disease
6. Capacity strengthening and engagement	Professional development opportunities for CSs to learn and apply skills for research as well as participate in policy activities; these can include in-service training, incentives, regular meetings to review evidence and determine recommendations, and strengthen relationships; all these increase the recognition and importance of evidence use in decision-making	*Enablers:* • Training programmes that connect senior MoH staff with young professionals in conducting and translating research into policy; in addition, programmes that equip CSs with the skills needed to conduct, analyse and disseminate research should provide opportunities for CSs to participate in policy activities where they can advocate for the use of and apply evidence • Opportunities for CSs to participate in policy activities where they interact with policy-makers and apply research skills • Strong links with academic institutions, providing more avenues for capacity-building
		*Barriers:* • Non-existence of such programmes due to different MoH priorities or lack of leaders’ support • CSs not knowing how to apply training in real policy-making • Often, training programmes do not cover enough or the appropriate skills and knowledge for evidence use (in particular related to synthesis and dissemination) and therefore training incentives and programmes do not necessarily lead to more evidence use • High turnover inhibits CSs from participating in trainings and applying skills
		*Illustrative examples (see Additional file for full descriptions):* • Sri Lanka’s Education, Training and Research Unit • South Africa’s Health Economic Unit

*CSs* Civil Servants, *DPs* Development Partners, *HPSR* health policy and systems research, *MoH* Ministry of Health

The first theme on leadership and political will came out clearly across all country case studies. The need for the governmental system as a whole to recognise the value of evidence in policy- and decision-making as well as the need for individual leaders to prioritise evidence generation and use and ensure that adequate motivation, funding and institutionalisation of processes is essential. Without strong skills in agenda-setting and management, when the health system focus is predominantly on service delivery, or when other political challenges arise, leaders can also contribute challenges and constraints to evidence uptake. A relevant example of this theme is the launch in Ethiopia’s Health Sector Development Program (HSDP) III in 2005. Within this programme, Ethiopia instituted the principle of ‘One Plan, One Budget, and One Report’ in order to harness the resources, expertise and evidence across multiple partners. The government of Ethiopia, at national and sub-national levels, was a key player in utilising evidence and leadership to instigate and carry out this national initiative, despite initial donor skepticism and push back.

Appropriate incentives and resources are also key to evidence uptake. These include both financial and non-financial incentives and resources, which can be developed and sustained through providing staff with opportunities such as networking with higher-level staff in their MoHs, travel to other institutions or countries to apply skills or observe other systems first-hand, salary top-ups related to research activities, and aligning government policies with international declarations and goals related to health spending and health-related research. Without resources or other incentives, such as career pathways, or when incentives unintentionally de-motivate staff that truly engage in problem-solving and evidence use, evidence uptake can be severely hindered. The history of Rwanda exemplifies this theme in a positive light. In the last 15 years, growth in financial resources paired with recognition of the need for an increase in capacity for evidence generation and use. The national response to these identified needs included a ‘district challenge fund’, a collaboration between the MoH and School of Public Health aiming to train district-level staff to analyse their own data and use research findings to make decisions.

Regarding infrastructure and access to health data, being able to access real-time data as well as share data across different agencies and partners are major enablers. Digitisation of datasets, which is underway across case study countries, can facilitate this as well. Lack of training on database use or dysfunctionality of these databases causes frustration and delays or failure to make use of existing data. In addition, language barriers or lack of communication processes within and between the MoH and other institutions, including universities and separate data or statistics units, and practices can limit access to or the usability of data. For example, the Chilean MoH identified the need for an integrated Health Technology Assessment process — one that centralises data collection, storage and access procedures to assist with research appraisal by multiple stakeholders. A central repository and established appraisal process via HTA would facilitate higher-quality evaluations of existing research before being used to inform policy decisions.

Designated structures and processes for evidence uptake were also identified across many of the cases. While ensuring that the system as a whole recognises the value of evidence uptake and engages in ensuring that it is happening, not every government agency or unit within a MoH is well-placed to nor needs to be fully responsible for handling the entire process on its own. Many countries found that establishing a designated unit within a MoH or across multiple sectors of government, including health, allowed for more focused and in-depth capacity to be built, provided a more coordinated platform for different partners to contribute, and enabled more coherent national research strategies and availability and use of related tools and resources. Without a designated structure, it was easy for governments to lose focus on evidence generation and use. In addition, when these structures did exist but lacked adequate funding or skills staff, these were also barriers. Well-designed structures for evidence use are exemplified in the Knowledge to Policy Center, a WHO collaborating center in Lebanon that is a key structure established to support the Ministry of Public Health as a capacity strengthening mechanism. Through its collaboration with the Knowledge to Policy Center, the Ministry of Public Health has co-facilitated a number of national policy dialogues that include key policy-makers, researchers and other stakeholders around high-priority issues in order to achieve policy impact.

In terms of interaction and relationships, having ongoing, structured and accountable contact between CSs, researchers and a range of other stakeholders was described as key to effectively utilising available capacity and different agencies’ strengths to support evidence uptake. In addition, international organisations as well as standards and best practices for collaboration and engagement can help overcome stalemates or barriers from ideological positions taken by any stakeholder. However, building such relationships and collaborations takes time and without facilitation or structured processes, communication channels and supportive leaders, these interactions can struggle or cease to function. In Kyrgyzstan, for example, in response to rising burden of cardiovascular disease, a process for developing an effective solution was not only informed by extensive research, but also by well-rounded interaction and inclusion of several stakeholders outside of the MoH. With the help of the involved agencies, intensive research served to create the National Strategy on Cardiovascular Disease Control, which in turn outlined policies improving the organisation of care and treatment as well as service delivery throughout the health system.

Finally, capacity strengthening and engagement of CSs was identified as another essential component for evidence uptake. This includes regular opportunities for early career and ongoing training and skill-building as well as initiatives and support for the establishment of mentorship models. Without leadership support and funding, such programmes are not possible. In addition, training programmes that do occur may not cover relevant skills, may not help learners link the concept learned in a training to actual policy-making processes, or turnover of CSs may result in skills gained not being able to be applied. The MoH of Sri Lanka has a strong example of this in its Education, Training and Research Unit, which functions as a hub for health personpower training as well as undertaking health research to inform the public sector health service delivery. The MoH has established training requirements in order to strengthen the capacity of CSs on topics related to evidence generation and uptake. Through the Education, Training and Research Unit’s research directorate, research allowances are available to academicians and senior level CS officers in order to motivate CSs to engage in research.

### Recommendations related to cross-cutting themes

We identified a set of key recommendations aligned with the six cross-cutting themes as well as specific actions that different stakeholders could undertake to further these recommendations. These recommendations are presented in Table 2. While most of the recommendations are focused on MoH roles and actions, there are number of things that other stakeholders, including academic institutions, development partners and civil society, can do to support or enhance evidence uptake. For example, in South Africa, researchers have been strong collaborators and advocates supporting the establishment of structures, committing to sustained engagement with MoH officials and working to ensure that findings from research are packaged and disseminated in formats that are accessible and useful to CSs [22]. The recommendations for MoHs and other stakeholders also often have the potential to be synergistic such as the recommendations under Theme 1. Here, the MoH has a key leadership role to plan in setting expectations and an example of evidence utilisation, and at the same time donors and academic institutions can support these processes by investing and helping to conduct research that aligns with national agendas and collaborate with MoH leadership.

**Table 2:** Recommendations and specific actions for each cross-cutting theme

Cross-cutting recommendation	Specific actions for different stakeholders
1. Leadership and political will: Support the MoH as a leader in setting a culture and example of evidence uptake in policy-making	• The MoH should continue to expand its leadership capacity and recognise itself as a critical leader and example setter for evidence use for the country • Donors can work to build institutional capacity by investing in research that supports national agendas and working in partnership with government • The MoH can build institutions for conducting and using research by creating roles in research, providing opportunities to advance to senior positions in research and recruiting more competent senior officials • The MoH should ensure a consistent and active presence with other agencies or research units (thematic/technical working groups, DPs, academics, etc.) to strengthen institutions for research uptake in policy debates • MoH leaders should prioritise professional development of CSs in order to improve incentives and thus capacity for research • Academic institutions and leaders can learn from each other and can champion evidence uptake • Academic institutions can ensure that the research, especially HPSR they generate, is demand driven and responds to the needs of national MoH and/or global public health priorities
2. Incentives and resources: Set targets for support for HPSR and other evidence generation and use, including domestic resources	• The MoH can review incentive structures and identify options to increase the retention of skilled CSs • National governments can dedicate a higher proportion of budget to health, science and research-related sectors • DPs can provide resources to test innovations and pilot test solutions but must feed the findings and experience back into national platforms and agendas in a supportive fashion
3. Infrastructure and access to data: Strengthen access to evidence, including routine data health information management systems and repositories of reports and other scholarly publications	• The MoH should continue to invest in data-sharing systems, including across sectors and efforts to digitise existing evidence • DPs should participate in national repositories and help facilitate the sharing of evidence that can inform policy
4. Designated structures and processes: Develop, maintain and utilise national research agendas and institutionalised units or centres whose focus is on evidence generation and use	• The MoH should lead a process to articulate a national research agenda that includes the voices of multiple stakeholders • The MoH can ensure that there is a focused entity that is functional and resourced and also responsible for supporting the implementation of that national agenda • Academic institutions should engage across the continuum of evidence use from problem identification to policy development/reform • DPs can work with and also provide support to the units or centres that are tasked with focusing on evidence generation and use
5. Interaction and relationships: CSs and researchers are key stakeholders to ensure that there is interactions and relationships. This is true on multiple levels, including sub-nationally, nationally and internationally	• The MoH can engage CSs at multiple levels in evidence-need identification, generation and use, which includes allocating time and resources to support them • Academic institutions should participate in structures set by the MoH to facilitate relationships and collaboration with CSs
6. Capacity strengthening and engagement: Continue to invest in CSs and other stakeholders through ongoing skill-building and engagement activities	• The MoH and other appropriate government and autonomous agencies should invest in providing formal and sufficient pathways for CS or aspiring health professionals to train in the skills needed to access data, conduct research, synthesise and disseminate findings to policy, programme managers and other stakeholders • The MoH should involve CSs in policy-related activities, such as applying research to write briefs, in order to motivate CSs to pursue research and strengthen institutions for research application and uptake processes

*CSs* Civil Servants, *DPs* Development Partners, *HPSR* health policy and systems research, *MoH* Ministry of Health

The recommendations we identified also build on successful actions and progress that have been seen across country contexts, and therefore it is an opportunity to further build on success, and certainly not an indication that efforts are starting from nothing. This is exemplified in Themes 4 and 5, where recommendations focus on MoHs maintaining a national agenda and sustaining a focused entity to support the implementation of that agenda, including the engagement of CSs throughout the process. The roles of academic institutions and development partners include engaging with these MoH-led structures and supporting the national agenda by collaborating across the continuum from problem identification to use of evidence to inform policy and practice. By taking a longer-term view of what capacity is desired and needed by a particular country, many stakeholders can be part of strengthening and facilitating greater evidence uptake by feeding into all of these recommendations.

## Discussion

By undertaking this framework analysis, we aimed to synthesise transferable and actionable lessons from across diverse contexts described in the nine country case studies. Each case represents a complex, constantly evolving process that we analysed looking at progress over time as well as areas for ongoing learning and further action. In addition, we recognise that case contexts continue to evolve and may have changed since the data presented in the case studies, or since the case studies were completed. We utilised country case examples as opportunities to learn from remarkable progress and also identify areas for continued work, and not to shed any country in a negative and punitive light.

We identified several limitations while undertaking the analysis for this paper. First, we were working with a set of case studies prepared by academic leaders in collaboration with highly experienced colleagues across all countries, which despite the use of a common ToC, did not have an equivalent level of detail or organisation of data on every aspect of the conceptual framework. We utilised rich descriptions from across countries and compared experiences across context when possible. We were not able to collect additional data in order to fill in identified gaps, as the case study development had already been completed and the approach utilised in developing and presenting the findings could not be completely standardised or quality controlled. In some instances, authors of the original cases also noted limitations in terms of not being able to access evidence across some aspects of the conceptual frameworks. In addition, the focus of this study as well as the original charge given to the nine country case study teams, was centered on institutional capacity for evidence uptake within government institutions. Perhaps partly due to this explicit focus from the outset, we did not find a lot of examples or descriptions of initiatives undertaken by or recommendations geared for other stakeholders such as researchers, donors and civil society. However, we know that, particularly within fields like HPSR, researchers have a strong responsibility and large potential for support and impact if they do engage proactively and collaborate with all stakeholders in ensuring evidence uptake [8, 23–25].

Finally, the recommendations gleaned from the case study analysis are general themes, which may not be relevant or timely in every context. Specific recommendations will likely require adaption and careful planning as well as strengthening of political alliance, establishing relationships and considering other contextual factors before implementation in a new setting.

## Conclusion

Evidence uptake has been an active pursuit for many countries, including the nine case countries analysed in this study. While each country has undertaken the process in different ways, common themes related to leadership, incentives and resources, infrastructure, designated structures, interaction, and capacity strengthening have been identified by comparing experiences from these diverse contexts. Continuing to strengthen capacity for evidence uptake in any country requires time, sustained investment on multiple levels, and active engagement of multiple stakeholders, including civil servants, researchers, development partners and civil society. Going forward, ongoing effort to strengthen both the supply and demand side of evidence uptake is needed across many countries; continuing to move this agenda forward will require the allocation of additional resources — particularly domestic resources — as well as further engagement of researchers to work alongside government leaders and civil servants throughout the evidence generation and uptake process.

## Supplementary information

**Additional file 1.**

## Data Availability

Data and materials utilised in this analysis are not publicly available as they were not commissioned with the purpose of being individually published. Interested readers may contact the Alliance for Health Policy and Systems Research if they wish to obtain further information about or materials from the country case studies.

## Abbreviations

CS: Civil servant
HPSR: Health policy and systems research
MoH: Ministry of Health
ToC: Theory of Change
